# ERA-CRISPR/Cas12a system: a rapid, highly sensitive and specific assay for *Mycobacterium tuberculosis*

**DOI:** 10.3389/fcimb.2024.1454076

**Published:** 2024-08-21

**Authors:** Tian Gan, Jianwei Yu, Zhongliang Deng, Jun He

**Affiliations:** ^1^ The Affiliated Nanhua Hospital, Department of Clinical Laboratory, Hengyang Medical School, University of South China, Hengyang, China; ^2^ Department of Public Health Laboratory Sciences, School of Public Health, Hengyang Medical School, University of South China, Hengyang, Hunan, China

**Keywords:** *Mycobacterium tuberculosis*, CRISPR/Cas12a, enzymatic recombinant isothermal amplification, fluorescence detection, lateral flow test

## Abstract

**Introduction:**

*Mycobacterium tuberculosis*, the causative agent of human tuberculosis, poses a significant threat to global public health and imposes a considerable burden on the economy. However, existing laboratory diagnostic methods for *M. tuberculosis* are time-consuming and have limited sensitivity levels.

**Methods:**

The CRISPR/Cas system, commonly known as the “gene scissors”, demonstrates remarkable specificity and efficient signal amplification capabilities. Enzymatic recombinase amplification (ERA) was utilized to rapidly amplify trace DNA fragments at a consistent temperature without relying on thermal cyclers. By integrating of CRISPR/Cas12a with ERA, we successfully developed an ERA-CRISPR/Cas12a detection system that enables rapid identification of *M. tuberculosis*.

**Results:**

The sensitivity of the ERA-CRISPR/Cas12a fluorescence and lateral flow systems was 9 copies/μL and 90 copies/μL, respectively. Simultaneously, the detection system exhibited no cross-reactivity with various of respiratory pathogens and non-tuberculosis mycobacteria, demonstrating a specificity of 100%. The positive concordance rate between the ERA-CRISPR/Cas12a fluorescence system and commercial qPCR was 100% in 60 clinical samples. Meanwhile, the lateral flow system showed a positive concordance rate of 93.8% when compared to commercial qPCR. Both methods demonstrated a negative concordance rate of 100%, and the test results can be obtained in 50 min at the earliest.

**Discussion:**

The ERA-CRISPR/Cas12a system offers a rapid, sensitive, and specific method that presents a novel approach to laboratory diagnosis of *M. tuberculosis*.

## 1 Introduction

*Mycobacterium tuberculosis* is a pathogen responsible for causing tuberculosis in humans. It was initially identified by German scientist Robert Koch in 1882. *M. tuberculosis* has the capability to infect various organs and tissues throughout the body, with pulrnonary tuberculosis being the most prevalent manifestation (Sia and Rengarajan, 2019; Rahlwes et al., 2023). The clinical symptoms of tuberculosis are intricate and diverse, particularly when it presents as extrapulmonary tuberculosis, leading to potential misdiagnosis or missed diagnosis by medical professionals. Therefore, enhancing the level of tuberculosis detection is crucial (Yang et al., 2023a). Currently, the etiological diagnosis of *M. tuberculosis* primarily relies on smear microscopy, isolation and culture techniques, serum antibody detection, and nucleic acid molecular detection methods (Uddin et al., 2019; Yang et al., 2023b). Smear microscopy offers quick results and simple operation but has a low detection rate. Isolation and culture of *M. tuberculosis* serve as the “gold standard” for diagnosing tuberculosis-related diseases; however, it grows slowly in Roche’s medium resulting in long experimental periods (Xiao et al., 2023). Colloidal gold-based assays are predominantly employed in laboratory settings for the detection of IgM and IgG antibodies against *M. tuberculosis* in serum or plasma, owing to their simplicity, convenience, and rapidity; however, these immunological methods exhibit limited sensitivity and accuracy due to inherent shortcomings associated with colloidal gold technology. Nucleic acid detection techniques mainly rely on PCR fluorescence probe technology for detecting *M. tuberculosis*, however, they require specialized laboratories equipped with professional technicians and equipment which hinders on-site point-of-care testing capabilities (Ji et al., 2020). Nevertheless, there still exist several limitations regarding time consumption, sensitivity, and portability within current laboratory tests for detecting *M. tuberculosis*. Therefore, it is imperative to develop a rapid, highly sensitive, and specific method for detecting *M. tuberculosis*.

Isothermal amplification technology, increasingly favored by scholars, eliminates the need for thermal cycling during nucleic acid amplification and exhibits minimal equipment requirements (Mota et al., 2022; Giantini et al., 2023). The Enzymatic Recombinase Amplification (ERA) technique is based on the principle of enzymatic recombinase combining with primers to form protein-DNA complexes that recognize homologous sequences in double-stranded DNA and initiate DNA synthesis (Li et al., 2023; Wang et al., 2023). ERA enables specific amplification of trace target DNA fragments within a constant temperature range of 37-42°C, demonstrating an impressively short reaction time of merely 15-20 min (Yang et al., 2021, 2022). With its highly specific tool enzyme, ERA reduces mismatches and test error rates significantly, rendering it applicable for detecting various genes such as the drug resistance gene FLT3-F691 in acute myeloid leukemia (AML), significantly improving AML diagnosis and treatment (Liu et al., 2021b). Moreover, ERA technology facilitates rapid and highly sensitive detection of shrimp liver and intestinal microsporidia, contributing to the control and prevention of shrimp parasitic diseases (Li et al., 2023).

**Figure 1:**
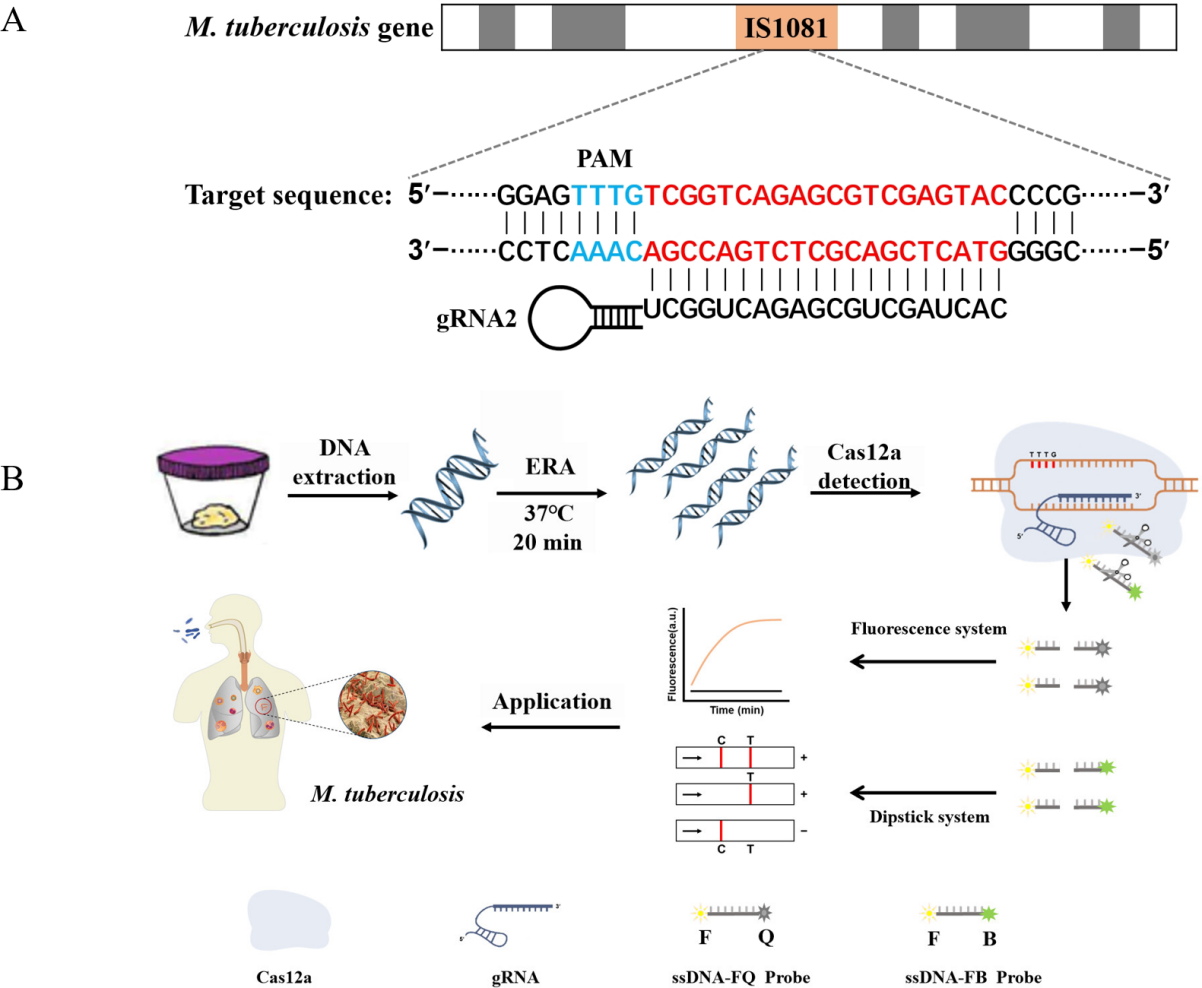
ERA-CRISPR/Cas12a schematic diagram. **(A)** The target gene sequence of *M. tuberculosis* H37Rv IS1081, including the protospacer adjacent motif (PAM) site and its corresponding reverse complementary fragment for guide RNA (gRNA) binding. **(B)** The working principles of the ERA-CRISPR/Cas12a dual detection system. DNA was extracted from a sample of *M. tuberculosis*. Following ERA amplification for 20 minutes, the LbCas12a-gRNA complex recognized the amplification product and initiated “collateral cleavage” activity, resulting in cleavage of the ssDNA-reporting probe. Qualitative detection of Mycobacterium tuberculosis was achieved through observation of fluorescence signal and side-flow strip. T, test line; C, control line.

CRISPR/Cas systems are prevalent in 40% of bacteria and 90% of archaea. These systems can be classified into two primary classes: Class 1 represents a multiprotein effector complex, while class 2 comprises a single Cas protein complex. Type II (Cas9), type V (Cas12a/b), and type VI (Cas13a/b) within the second category have emerged as widely used genome editing tools. The Cas12a protein, also known as Cpf1, consists of approximately 1200-1300 amino acids and functions as an endonuclease guided by a single RNA molecule (Hendriks et al., 2020; Khan and Sallard, 2023). By combining Cas12a ssDNase activation with isothermal amplification techniques, the Doudna group has developed DETECTR, a method enabling detection with atomic molar sensitivity (Chen et al., 2018). The underlying principle involves crRNA binding to Cas12a protein to form a Cas-crRNA complex that recognizes the T-rich protospacer adjacent motif (PAM). Subsequently, crRNA binds to guide-complementary ssDNA through base pairing interactions, resulting in robust release of nonspecific ssDNA trans-cleavage activity (Tian et al., 2022; Peng et al., 2024). Zhang et al. demonstrated that the ERA-CRISPR/Cas12a reaction is capable of detecting PCV3 in genomic DNA with a minimum of seven copies within one hour (Zhang et al., 2021). Furthermore, our research group employed the ERA-CRISPR/Cas12a technique for rapid detection of *M. pneumoniae* (Deng et al., 2022). CRISPR/Cas is currently recognized for its significant potential in TB diagnosis and may emerge as a viable approach for TB eradication in the future (Shi et al., 2024). Therefore, this study aims to leverage the advantages of the ERA-CRISPR/Cas12a system’s speediness, ultra-sensitivity, and high specificity to establish an efficient laboratory method for *M. tuberculosis* (**Figure 1**).

## 2 Materials and methods

### 2.1 Materials and reagents

The ERA Basic Kit (No. KS101) and lateral flow test strip (NO.TS104) were procured from Suzhou GenDx Biotech Co., LTD. (China). LbCas12a recombinant protein (NO. E-002) was obtained from Shanghai HuicH Biotech Co., LTD. (China). The commercial qPCR kit was purchased from Hunan Sansure Biotech Co., LTD. (China). NEBuffer 2.1 Buffer was sourced from New England BioLabs (No. B7202). The oligonucleotide and recombinant plasmid were chemically synthesized by Shanghai Sangon Biotechnology Co. (China).

### 2.2 Strains and clinical samples

The *M. tuberculosis* H37Rv strain (ATCC 27294) was obtained from the Laboratory Department of the Affiliated Changsha Central Hospital of University of South China. Our research group has successfully preserved the strains of *Mycoplasma pneumoniae* M129 (ATCC 29342), *Staphylococcus aureus* (ATCC 25923), and *Pseudomonas aeruginosa* (ATCC 27853). The *Mycobacterium kansassi* strain (ATCC 12478), *Mycobacterium fortuitum* (ATCC 6841), *Mycobacterium avium* (ATCC 25291), *Mycobacterium asiaticum* (ATCC 25274), *Mycobacterium gordona* (ATCC 14470), and *Mycobacterium chelonae* (ATCC 35752) were donated by Hunan Sansure Biotech Co., Ltd. The clinical samples used in this study were obtained from the Laboratory Department of the Affiliated Nanhua Hospital of University of South China. The collection and processing of all strains and clinical samples strictly adhered to the recommended procedures of the World Health Organization and the Laboratory Department of the Affiliated Nanhua Hospital, which were conducted in a BSL-2 laboratory for DNA extraction. This study has undergone review and approval by the Ethics Committee of the Affiliated Nanhua Hospital of University of South China (2023-KY-04).

### 2.3 Design of ERA primers

The IS1081 gene, which contains 5-7 repetitive sequences, is universally present in all *M. tuberculosis*. This genetic marker plays a crucial role in the accurate and specific diagnosis of *M. tuberculosis* (Lyu et al., 2020). In this study, we have confirmed the remarkable conservation of the IS1081 sequence (NC_000962.3: 3381351-3382674) through alignment using the online bioinformatics software BLAST (National Center for Biotechnology Information, NCBI). Following ERA primer design principles, we utilized Prime-BLAST (NCBI) online software to design primers and evaluate their specificity to ensure absence of mismatches with other pathogens (**Table 1**).

**Table 1:** Primers and probes used in the ERA-CRISPR/Cas12a system.

Name	Sequence (5’-3’)	Length
Forward Primer 1 (F1) *	GTCCTTCGATCCATTCGTCGTGTTGTTCGG	30
Forward Primer 2 (F2)	CTTCGATCCATTCGTCGTGTTGTTCGG	27
Reverse Primer 1 (R1) *	GAATCAGTTGTTGCCCAATATGATCGGGTACT	32
Reverse Primer 2 (R2)	CAATATGATCGGGTACTCGACGCTCTGACC	30
F-Q probe	5´-FAM-TTATTATT-BHQ-3´	8
F-B probe	5´-FAM-TTATTATT-Biotin-3´	8

F and R were the forward and reverse primers of ERA. The final sequencesare marked with an asterisk (*).

### 2.4 Design of gRNA and reporter probes

A mature gRNA typically consists of 19-nucleotide repeats, followed by intervals of 23-25 nucleotides. The specific repeat sequence is 5’-UAAUUUCUACUAAGUGUAGAU-3’. Positioned downstream of the TTTN PAM site, the spacer sequence perfectly matches with the target gene. To identify a suitable gRNA for *M. tuberculosis*, we employed CRISPR-DT, an online software tool based on Cpf1 technology that predicts both cutting efficiency and potential off-target effects. This analysis led us to select the most optimal gRNA as outlined in **Table 2**.

**Table 2:** The sequence of gRNAs used in this study.

Name	Sequence (5’-3’)
gRNA1	UAAUUUCUACUAAGUGUAGA UCGCGGUCUAGACGAACCCCU
gRNA2*	UAAUUUCUACUAAGUGUAGAUU CGGUCAGAGCGUCGAGUAC

The final sequence of gRNA used in the MTB Cas12a fluorescence assay (*).

After activation, the LbCas12a protein exhibits “Collateral cutting activity”. Therefore, we utilized this activity to design a fluorescent probe called oligonucleotide (F-Q), which is a non-specifically cleaved single-stranded DNA labeled with FAM fluorescence dye at the 5’ end and BHQ1 quenching group at the 3’ end. Subsequently, changes in fluorescence intensity were monitored. For the lateral flow reporter probe, we selected the F-B probe with the sequence 5’-FAM and 3’-Biotin.

### 2.5 Establishment and optimization of the ERA-CRISPR/Cas12a fluorescence system

The ERA-CRISPR/Cas12a system was constructed in this study through a two-step process. In the initial step, the Basic ERA kit was utilized for isothermal amplification of the IS1801 gene fragment of *M. tuberculosis*. A total volume of 48 µL ERA mixture was prepared by adding 20 µL of solvent, 2.5 µL each of forward and reverse primers (10 µM), 21 µL of ddH_2_O, and 2 µL of template in a reaction tube. Subsequently, the 48 µL mixture was transferred to a reaction tube containing ERA lyophilized powder, thoroughly mixed, and then added with 2 µL ERA activator on top of the tube cover before tightly sealing it and briefly centrifuging it. The reaction tube was incubated at 37°C for 20 min. In the second step, adding a volume of 2 µL amplification product to a 100 µL reaction mixture containing gRNA, Cas12a, F-Q fluorescent probe, and NEBuffer 2.1 (final concentration:1×) for CRISPR/Cas12a detection purposes. The fluorescence signals were continuously monitored for 40 min at 37°C using an ABI7500 qPCR instrument (Thermo Fisher, Massachusetts).

In order to determine the optimal experimental conditions, a comprehensive exploration of the entire system was conducted. This included screening gRNA, selecting ERA primers, optimizing ERA reaction temperature, gRNA concentration, LbCas12a concentration, and F-Q concentration. Firstly, we screened compatible gRNA for the target gene and subsequently paired the primers into four pairs to identify the most optimal ones. Secondly, by observing the impact of different temperatures on ERA reaction performance, we determined the optimal reaction temperature for ERA. Additionally, we investigated the concentrations of gRNA and LbCas12a protein to establish the ideal complex concentration for binding between LbCas12a and gRNA. Furthermore, we clarified the optimum working concentration for branch cutting F-Q reporter probes. Finally, this study examined the amount of added ERA amplification products in order to ascertain the best reaction efficiency for two-step experiments.

### 2.6 Establishment and optimization of the ERA-CRISPR/Cas12a lateral flow system

The ERA-CRISPR/Cas12a lateral flow assay was developed based on a fluorescence-based platform, differing only in terms of CRISPR probe selection and incubation duration. Within this novel approach utilizing a lateral flow format, an alternative F-B probe replaces F-Q as part of our experimental design. The CRISPR/Cas12a reverse cleavage reaction is conducted at 37°C for 15 min in a volume of 100 µL. Subsequently, 50 µL of CRISPR/Cas12a solution is added to the sample area of the lateral flow strip, and positive results can be observed within 5 min. The concentration of the F-B report probe and the incubation time play crucial roles in reading results on lateral flow test strips in the lateral flow detection system. To optimize this system, this study investigated the detection efficiency of the F-B probe at various concentrations (ranging from 50 nM to 800 nM) and incubation times with CRISPR/Cas12a (ranging from 5 min to 30 min).

### 2.7 Assessment of the sensitivity and specificity of ERA-CRISPR/Cas12a dual system

The pUC57-IS1081 recombinant plasmid was constructed by cloning the target segment of IS1081 into the pUC57 plasmid. To assess the sensitivity of this detection system, a 10-fold concentration gradient (10^5^-10^0^ copies/µL) of the recombinant plasmid was prepared as amplification templates. The formula for determining the number of DNA molecule copies is as follows: (Copies/μL) = [DNA concentration (g/μL)/(plasmid base length × 660)] × 6.02 × 10^23^. Additionally, we also incorporated controls for identifying 9 prevalent respiratory pathogens, including 3 common respiratory pathogens (*Staphylococcus aureus*, *Pseudomonas aeruginosa*, *and Mycoplasma pneumoniae*), as well as 6 NTM (non-tuberculous mycobacteria) strains to evaluate the specificity.

### 2.8 Clinical samples analysis

The sputum samples were treated with a four-fold volume of 4% NaOH solution for complete liquefaction (approximately 30 min), followed by DNA extraction. To evaluate the concordance rate of the ERA-CRISPR/Cas12a dual system in clinical specimens, each sputum sample was subjected to both the ERA-CRISPR/Cas12a dual detection system and qPCR. Enzyme-free water was utilized as a negative control. The positive or negative predictive value (PPV/NPV) of the ERA-CRISPR/Cas12a-dual system was evaluated by comparing it to qPCR results, which served as the reference standard.

The DNA extracted was amplified through real-time PCR utilizing a commercially available qPCR kit, and the results were monitored using an ABI 7500. PCR-mix was prepared by combining 38 μL of reaction solution, 2 µL of enzyme mixture, and 1 µL of internal standard. Subsequently, a 5 μL DNA template was added. The thermal cycling program is as follows: 50°C for 2 min, 94°C for 5 min, 45 cycles were performed at 94°C for 15 s and 57°C for 31 s, and finally, cooling down to 25°C for 10 s.

## 3 Results

### 3.1 Feasibility analysis for the ERA-CRISPR/Cas12a fluorescence system

**Figure 2:**
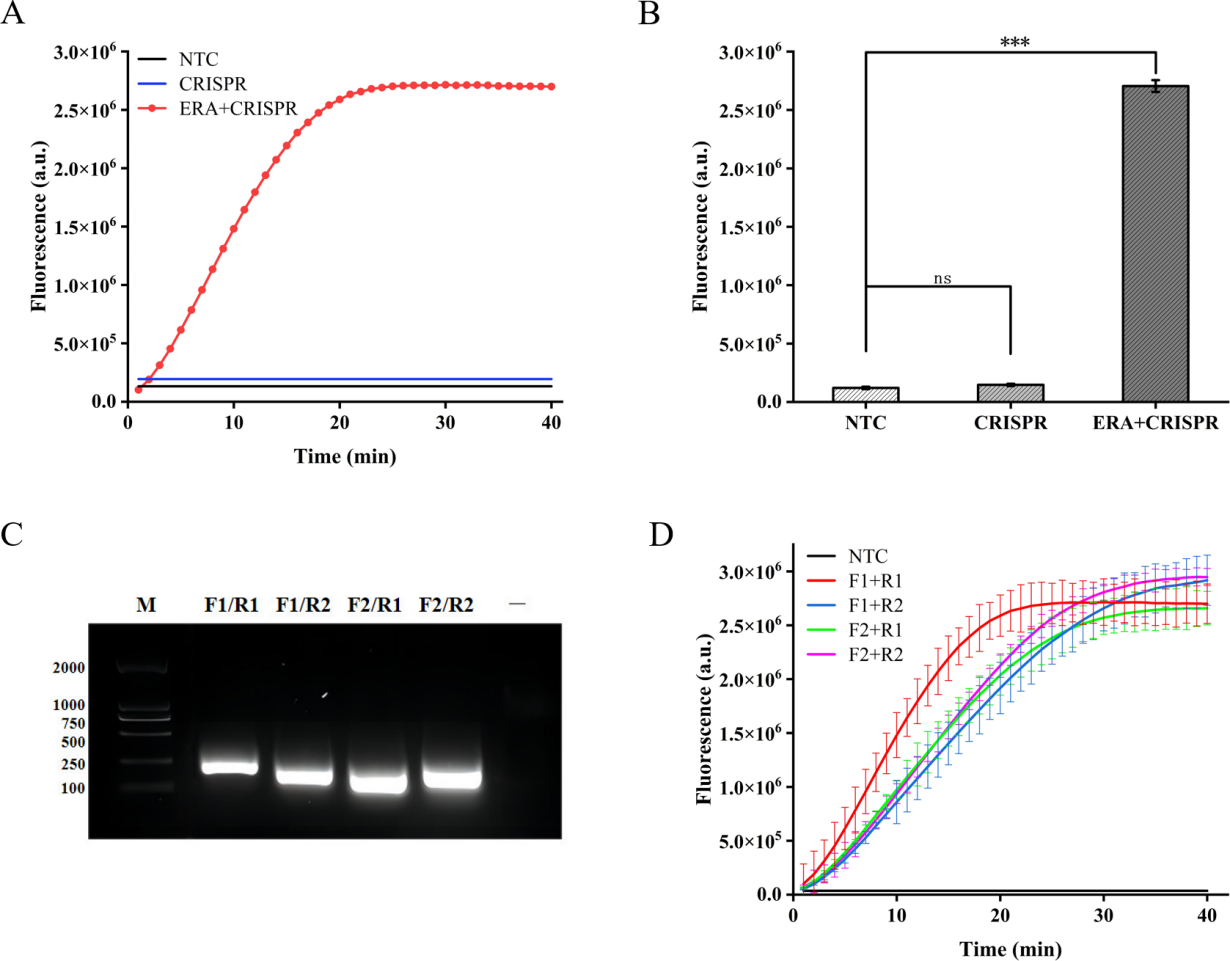
Feasibility analysis of the ERA-CRISPR/Cas12a fluorescence system and ERA primers screening. **(A, B)** Feasibility analysis of ERA amplification technology combined with CRISPR/Cas12a system. NTC: negative control. The components of the reactants are identical to those in the test tube, with the sole distinction being the replacement of the template with enzyme-free water. CRISPR: samples not amplified by ERA are directly detected using the CRISPR system. ERA + CRISPR: detection results of the entire reaction system. **(C)** After ERA amplification with four sets of cross-primers, the amplified products were electrophoresed in a 1.5% (w/v) agarose gel at 120 V for 30 minutes. M, DNA Marker; —, The sample only includes Loading Buffer. **(D)** The optimal primer pairs for H37Rv IS1081 were screened. F1, forward primer1; R1, reverse primer 1; F2, forward primer 2; R2, reverse primer 2. Error bars in panels represent the mean ± SD, where n=3 replicates. ***p<0.001. ns, no significance.

We initially assessed the applicability of the ERA-CRISPR/Cas12a system for detecting the *M. tuberculosis* IS1081 gene. The experimental results revealed that during the initial stage of the reaction, there was a rapid increase in fluorescence intensity within a short time period, followed by stabilization at approximately 20 min with a fluorescence value reaching up to 2.7×10^6^ (arbitrary units). This value was significantly different from that of the control group (*p* < 0.001) (**Figures 2A, B**). These findings suggest that the ERA-CRISPR/Cas12a system exhibits fast and achieves saturation within 20 min. Furthermore, it was observed that this system accurately and rapidly identifies the *M. tuberculosis* IS1081 gene while initiating collateral cutting activity upon gRNA-Cas12a complex activation. Notably, when performing CRISPR/Cas12a reactions were conducted without ERA amplification, no significant change in fluorescence intensity was observed compared to the control group (*p* > 0.05). These results underscore the feasibility of integrating ERA with the CRISPR/Cas12a system.

### 3.2 Optimization of the ERA amplification

In this study, we designed two pairs of primers targeting the highly conserved IS1081 gene in *M. tuberculosis*. By combining these two pairs of primers, four different primer combinations were generated. Agarose gel electrophoresis analysis confirmed the exceptional specificity of all four primer combinations during amplification (**Figure 2C**). In order to further validate the optimal amplification efficiency of each primer pair in the ERA-CRISPR/Cas12a system, verification was conducted using the ERA-CRISPR/Cas12a fluorescence system with four pairs of primers. The experimental results demonstrated that the combination of forward primer 1 and reverse primer 1 (F1+R1) exhibited the highest amplification efficiency and was confirmed as the most effective reaction primer pair (**Figure 2D**).

**Figure 3:**
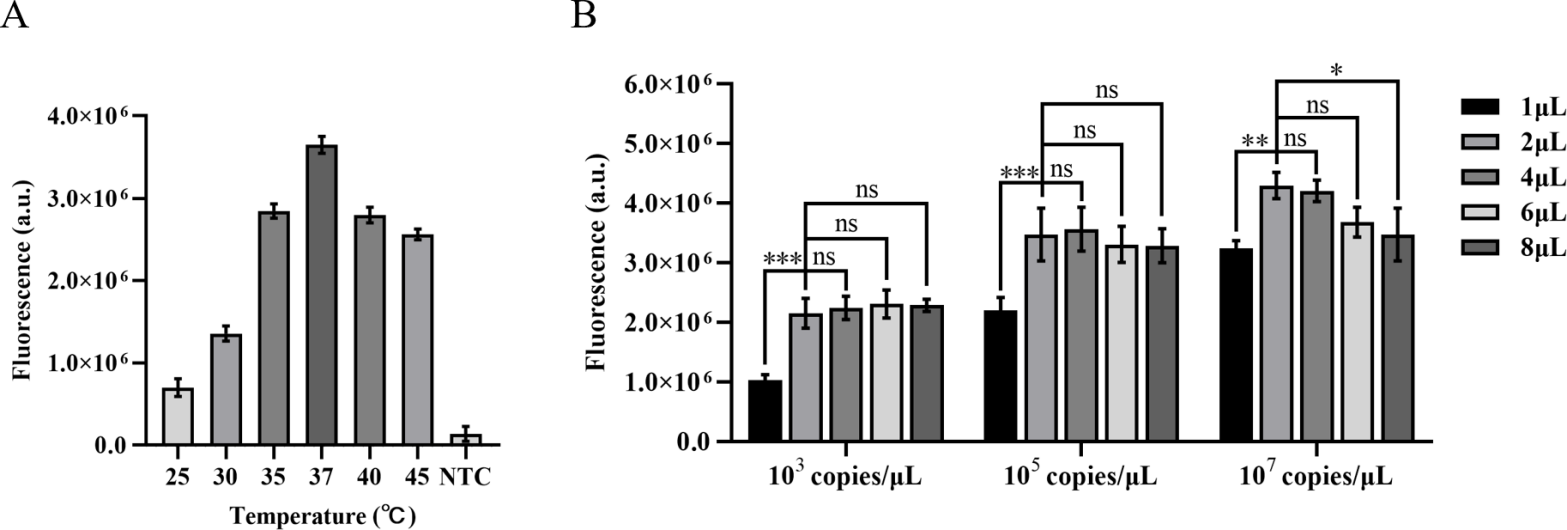
Optimization of ERA amplification temperature and the quantity of the ERA product. **(A)** Optimization of the ERA amplification temperature. **(B)** The impact of varying concentrations of H37Rv gene DNA templates and different amounts of ERA products on the CRISPR/Cas12a cleavage reaction. Error bars in panels represent the mean ± SD, where n=3 replicates. ***p<0.001. **p<0.01. *p<0.05. ns, no significance.

The amplification efficiency gradually improves with the increase in temperature during ERA amplification, reaching its peak fluorescence intensity at 37°C. Comparatively, the amplification effect at 40°C is equivalent to that at 35°C. Based on these findings, this study designates 37°C as the optimal temperature for ERA-CRISPR/Cas12a amplification (**Figure 3A**).

The high concentrations of template DNA may result in crowding effects, disrupting the balance within the reaction system and thereby inhibiting the CRISPR cleavage reaction. Due to the unknown concentration of template in clinical samples, we diluted *M. tuberculosis* H37Rv genomic DNA to 10^3^ copies/µL, 10^5^ copies/µL, and 10^7^ copies/µL in order to minimize this interference. We compared the impact of different template concentrations on ERA amplification products in the CRISPR cleavage reaction for application to samples with unknown concentrations. The results indicated that an increase in the quantity of ERA product led to a gradual rise in fluorescence levels at template concentrations of 10^3^ copies/µL and 10^5^ copies/µL. The highest fluorescence intensity was observed at a volume of 4 µL, with no significant difference between volumes of 2 µL and 4 µL (*P*<0.05). At a template concentration of 10^7^ copies/µL, the maximum fluorescence intensity was observed at a volume of 2 µL. Therefore, we opted to add 2 µL of ERA amplification product (**Figure 3B**).

### 3.3 Optimization of the CRISPR/Cas12a fluorescence system

**Figure 4:**
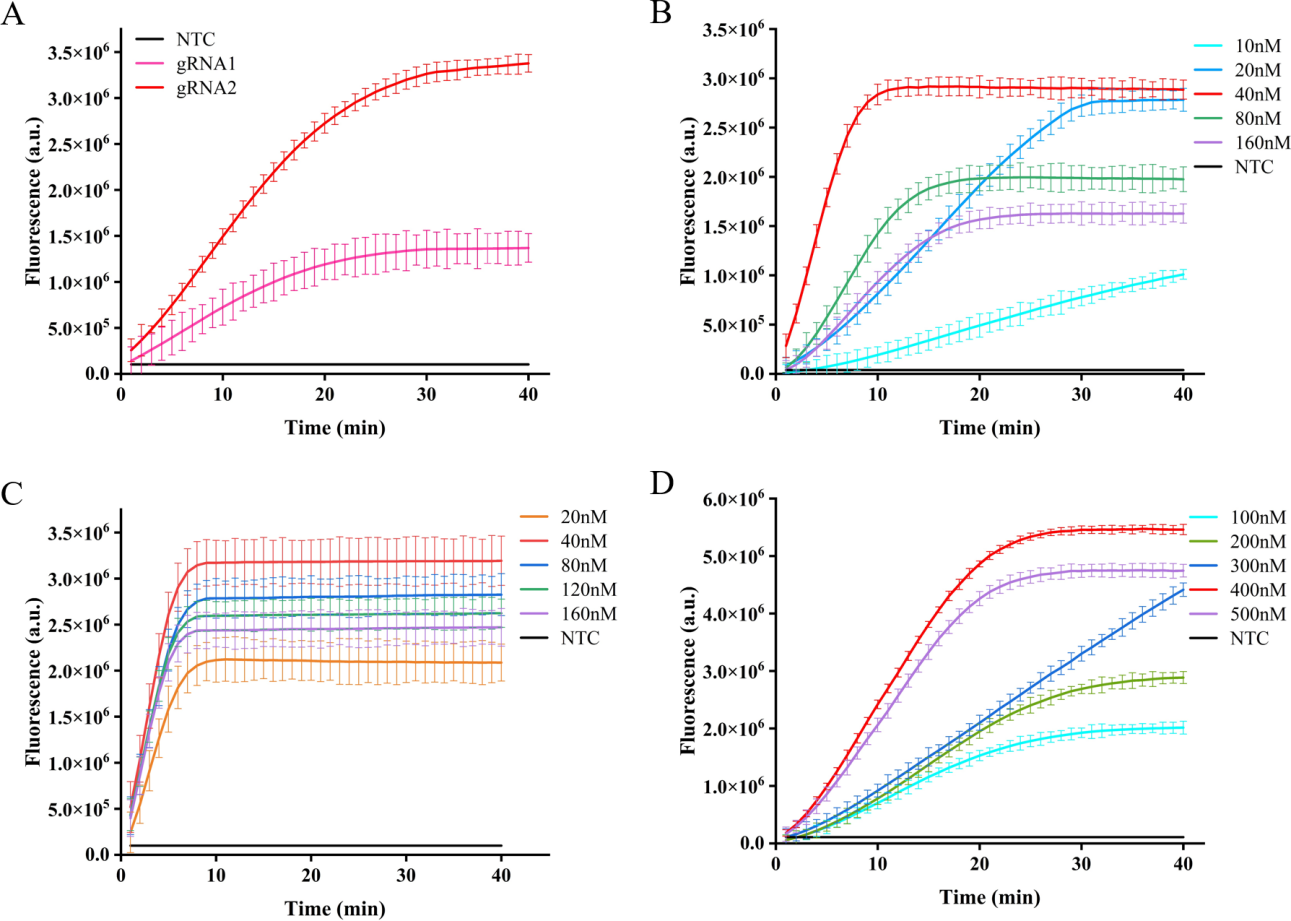
Optimization of CRISPR-Cas12a system in ERA-CRISPR/Cas12a fluorescence system. The fluorescence intensity of the system was optimized for gRNA screening **(A)**, gRNA concentration optimization **(B)**, LbCas12a concentration optimization **(C)**, and F-Q probe concentration optimization **(D)**. Error bars in panels represent the mean ± SD, where n=3 replicates.

Different gRNA-Cas12a protein complexes may exhibit varying levels of collateral cutting activity. In this investigation, two gRNAs were designed to target the IS1081 gene. The findings revealed that gRNA2 demonstrated a more pronounced fluorescence curve and higher fluorescence intensity compared to gRNA1 at 40 min, the maximum fluorescence intensity generated by gRNA2 was approximately 2.5 times greater than that of gRNA1 and roughly 12 times higher than the control group, demonstrating the superior performance of gRNA2 (**Figure 4A**).

After selecting the optimal gRNA, we investigated and optimized the concentrations of gRNA, LbCas12a protein, and F-Q probe in the CRISPR/Cas12a system. By comparing the fluorescence curve intensities generated by different concentrations of gRNA and LbCas12a protein, we observed that the CRISPR reaction reached a plateau phase at approximately 10 min with a gRNA concentration of 40 nM. Moreover, when maintaining an LbCas12a protein concentration of 40 nM, the fluorescence intensity peaked (**Figures 4B, C**). Subsequently, the reaction system was supplemented with varying concentrations of fluorescent reporter probes. It was observed that equilibrium was achieved within a span of 20 min at a concentration of 400 nM, leading to the attainment of maximum fluorescence intensity (**Figure 4D**).

### 3.4 Optimization of the CRISPR/Cas12a lateral flow system

**Figure 5:**
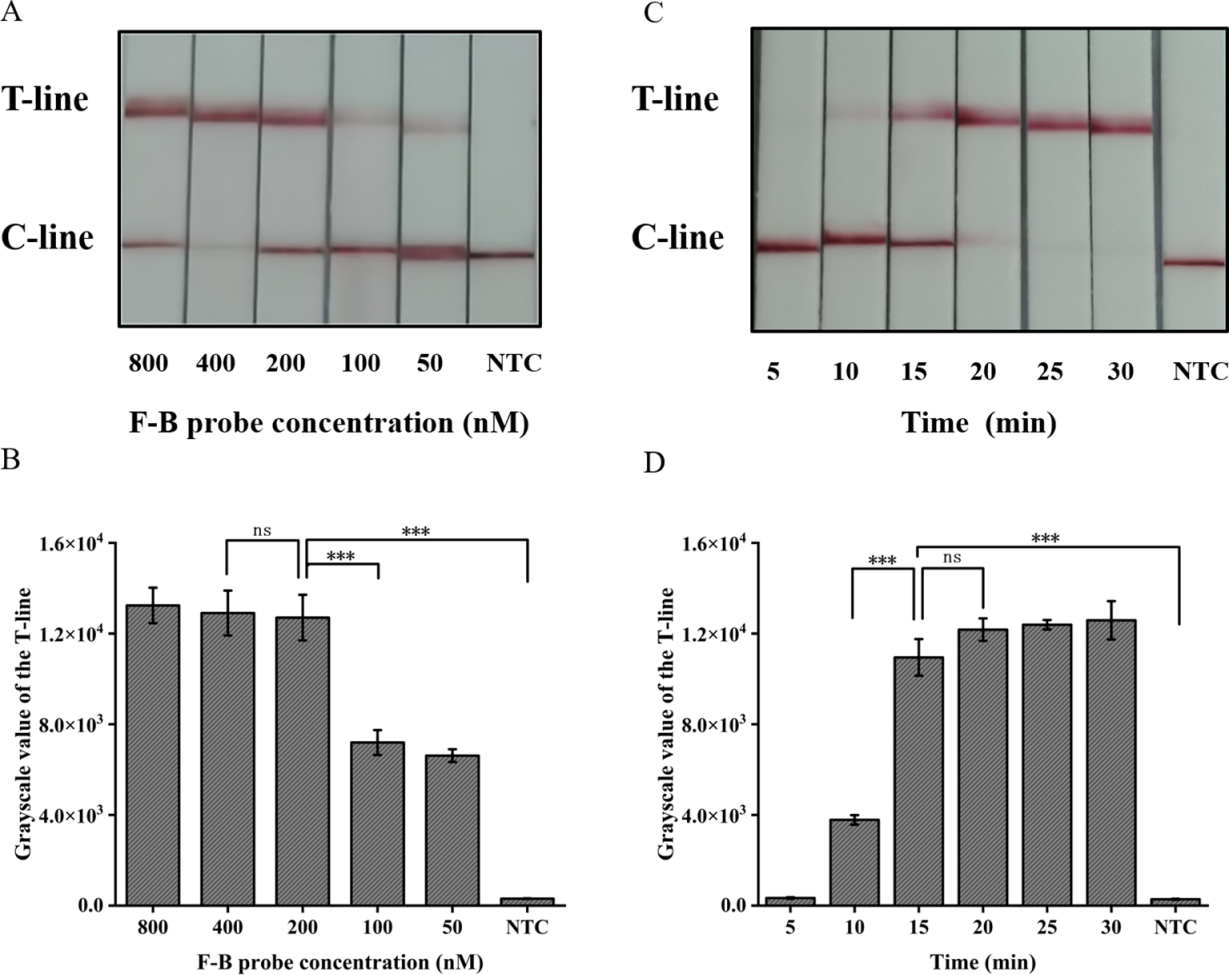
Optimization of the CRISPR/Cas12a lateral flow system in ERA-CRISPR/Cas12a. **(A)** Optimization of the F-B probe concentration and **(B)** the grayscale scanning result of the T line. **(C)** Optimization of CRISPR/Cas12a reaction time and **(D)** the grayscale scanning result of the T line. Error bars in panels represent the mean ± SD, where n=3 replicates. T, test line; C, control line. ***, P<0.001; ns, no significance.

The absence of the T-line on the lateral flow band indicates a negative result; however, only the presence of the T-line can be interpreted as a positive outcome. To minimize errors caused by subjective factors, this study utilized gray scanning of T-lines on the transverse flow bar, with the gray value of negative control T-lines set at three times the threshold for interpretation results. Analyzing the results, we considered that the concentration of the probe plays a crucial role in ensuring accurate readings. The results indicate that a minimum concentration of 200 nM for the F-B probe is required in order to clearly observe the T-line (**Figures 5A, B**).

The present study further investigated the impact of incubation time on the lateral flow detection system utilizing CRISPR/Cas12a. Incubations were performed at 37°C for durations of 5, 10, 15, 20, 25, 30 min respectively. As shown in **Figures 5C, D**, the T-line can be clearly observed after an incubation period of at least 15 min. Consequently, the optimal duration for incubation in the test paper system was determined to be 15 min.

### 3.5 Sensitivity and specificity validation of the ERA-CRISPR/Cas12a dual system

**Figure 6:**
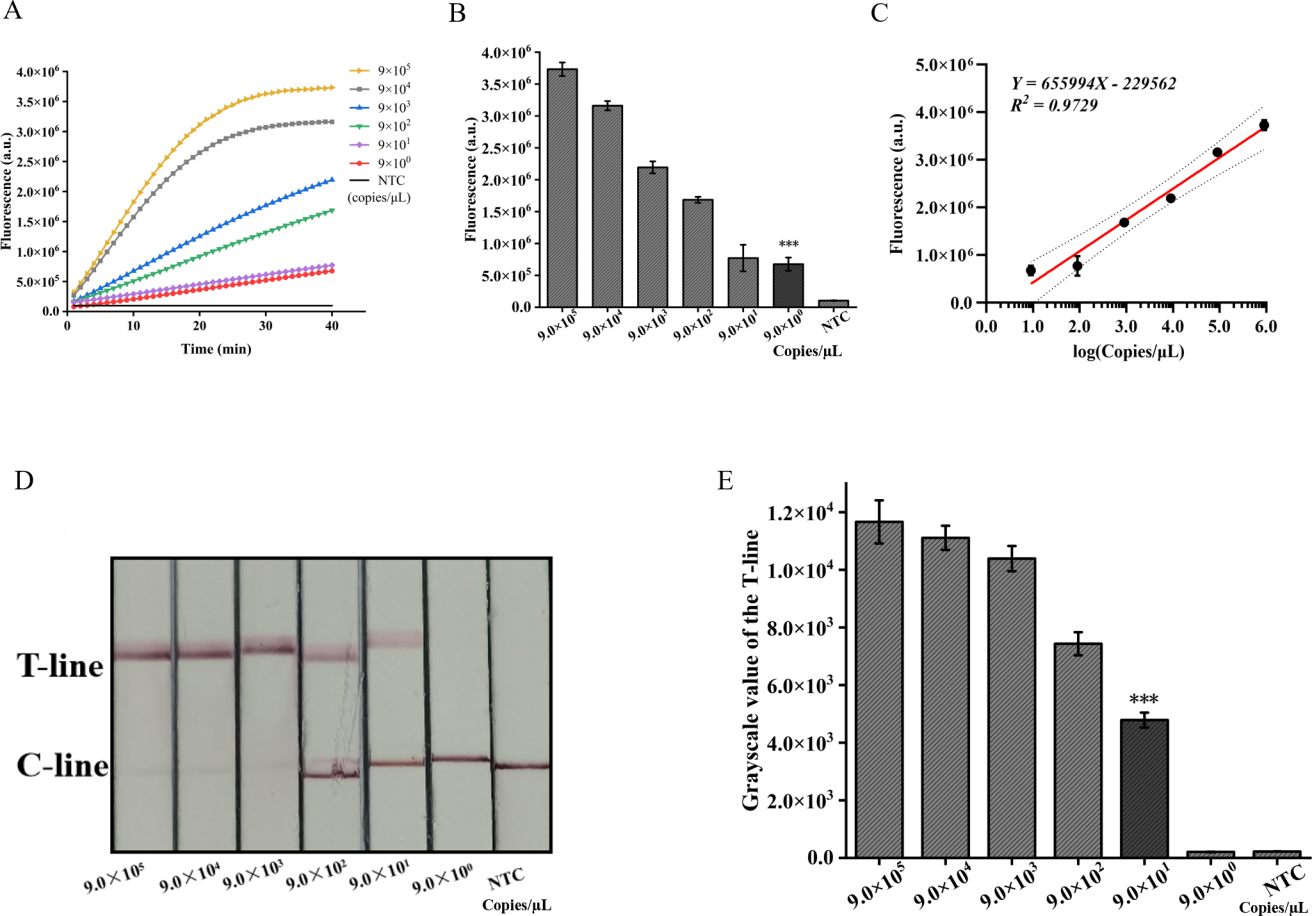
Validation of sensitivity of ERA-CRISPR/Cas12a dual detection system. **(A)** Fluorescence curves generated by the ERA-CRISPR/Cas12a fluorescence system at various target concentrations. **(B)** Comparison of fluorescence values generated after 40 min of ERA/CRISPR-Cas12a fluorescence system. **(C)** The calibration plots of fluorescence intensity versus the logarithm of target concentration. **(D)** Sensitivity analysis of ERA/CRISPR-Cas12a lateral flow system at various target concentrations and **(E)** the grayscale scanning result of the T line. Error bars in panels represent the mean ± SD, where n=3 replicates. T, test line; C, control line. ***, P<0.001.

The pUC57-IS1081 plasmid served as a template for dilution within a range from 9.0×10^5^ to 9.0×10^0^ copies/µL. The findings revealed a proportional reduction in fluorescence accumulation with decreasing IS1081 plasmid concentration. The limit of detection (LOD) for the ERA-CRISPR/Cas12a fluorescence detection system was established at 9 copies/µL, whereas that for the lateral flow detection system stood at 90 copies/µL (**Figures 6A, B, D, E**). Notably sensitive, the fluorescence-based method demonstrated linearity between fluorescent signal and the logarithm of the target concentration with an equation *Y=655994X -229562*, *R^2 ^= 0.9729* (**Figure 6C**), making it particularly suitable for semi-quantitative *M. tuberculosis* DNA analysis at low concentrations. Despite its comparatively lower sensitivity, the lateral flow assay offers outstanding portability and is well-suited for use in resource-constrained environments.

**Figure 7:**
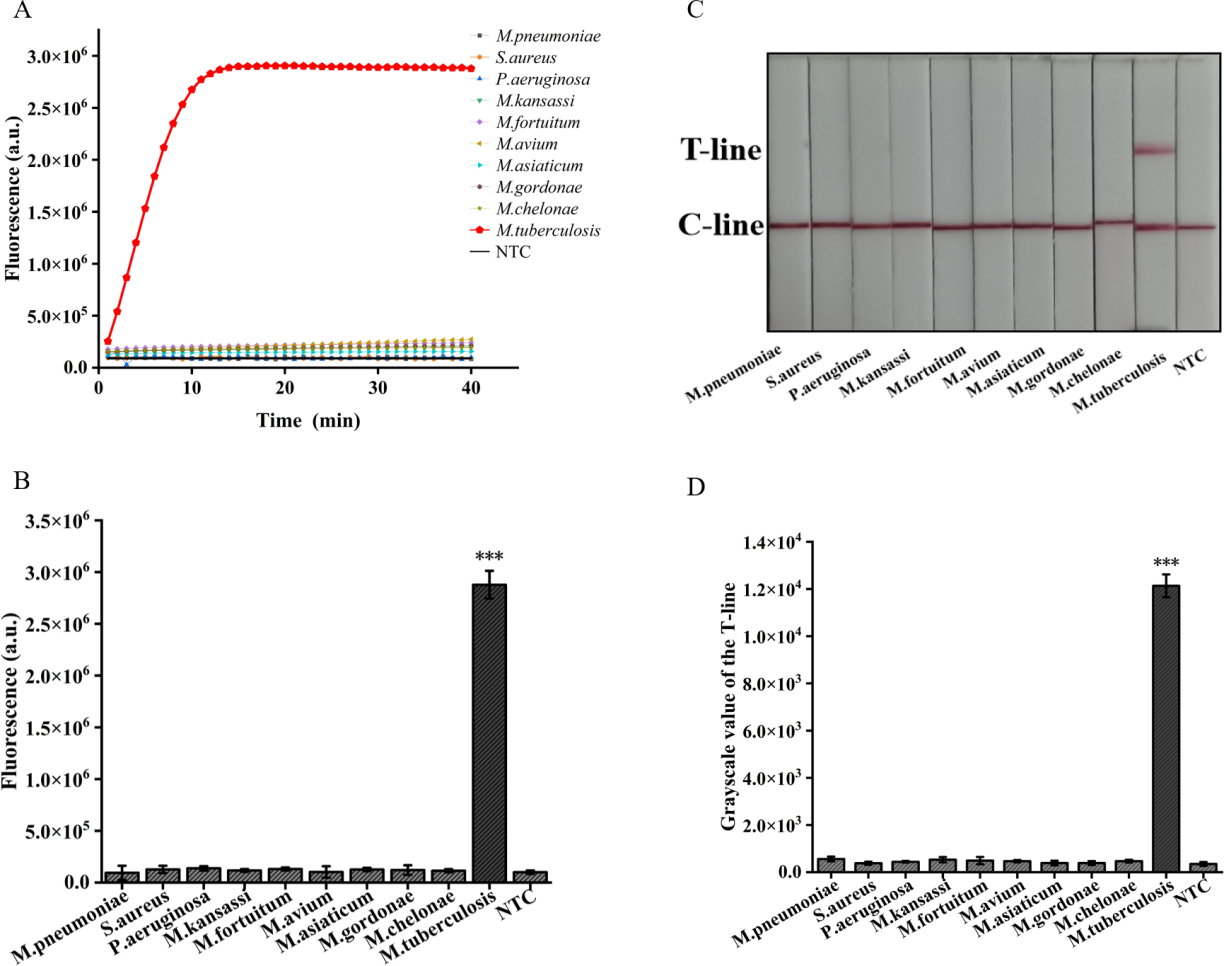
Validation of Specificity of ERA-CRISPR/Cas12a dual detection system. **(A)** Specificity of the ERA/CRISPR-Cas12a fluorescence detection system for 3 respiratory pathogens and 6 NTM strains. **(B)** Comparison of fluorescence values generated after 40 min of ERA/CRISPR-Cas12a fluorescence system. **(C)** Specificity of the ERA-CRISPR/Cas12a lateral flow system for 3 respiratory pathogens and 6 NTM strains and **(D)** the grayscale scanning result of the T line. Error bars in panels represent the mean ± SD, where n=3 replicates. T, test line; C, control line. ***p<0.001.

In addition, we employed two detection systems to examine the presence of three common respiratory tract infection pathogens and six NTM strains. The outcomes obtained from the fluorescence detection system revealed a statistically significant distinction solely between *M. tuberculosis* and the negative control (*p*<0.001) (**Figures 7A, B**). Furthermore, the results derived from lateral flow detection also demonstrated that only *M. tuberculosis* exhibited a distinct T-line (**Figures 7C, D**). The research findings demonstrate that both detection systems exhibit no cross-reactivity with other pathogens, thereby attaining a specificity of 100%. In conclusion, the ERA-CRISPR/Cas12a dual detection system exhibits remarkable sensitivity and specificity, thus holding promising prospects for clinical sample testing.

### 3.6 Applicability of ERA-CRISPR/Cas12a dual system assay to clinical samples

**Figure 8:**
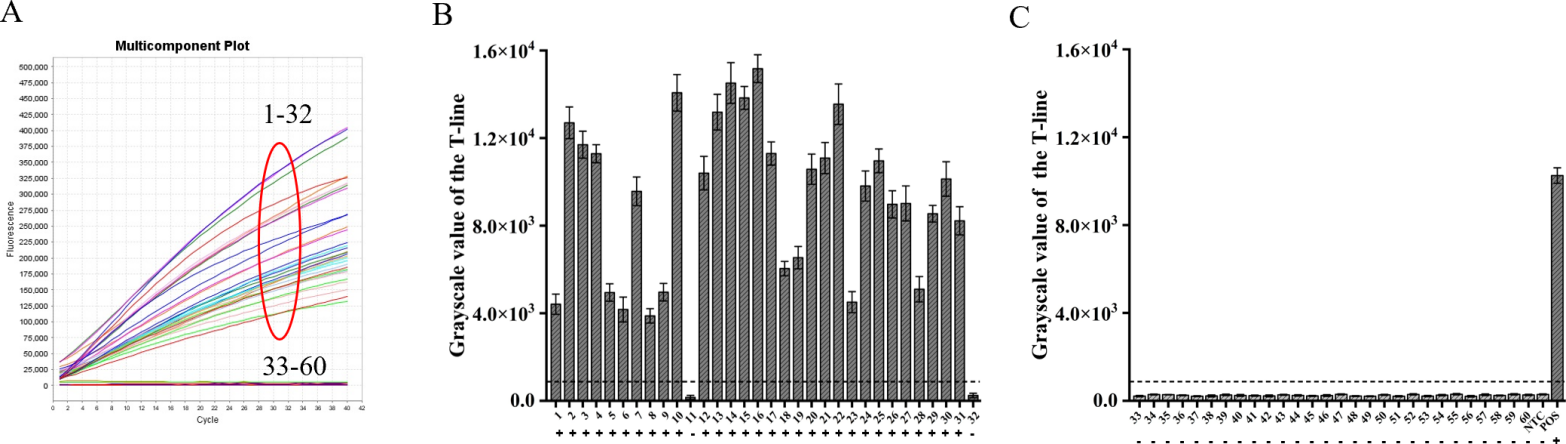
Verification of ERA-CRISPR/Cas12a dual system using clinical samples. **(A)** Detection results of the ERA-CRISPR/Cas12a fluorescence system. **(B, C)** The grayscale scanning results of the T line in the ERA-CRISPR/Cas12a lateral flow system. +, indicates a positive sample; −, indicates a negative sample; NTC, negative control; POS, positive control. Error bars in panels represent the mean ± SD, where n=3 replicates.

By utilizing the ERA-CRISPR/Cas12a fluorescence detection system, a total of 60 clinical samples were successfully validated. Samples exhibiting a fluorescence value to negative control ratio exceeding three times were designated as positive samples (Ding et al., 2021). According to the results, the ERA-CRISPR/Cas12a fluorescence and lateral flow systems demonstrated a positive predictive value of 100% and 93.8%, respectively, when compared to commercial qPCR detection. Both systems exhibited a negative predictive value of 100% (**Table 3**; **Figures 8A–C**). These findings suggest that this testing system is comparable to commercially available qPCR reagent kits in terms of clinical sample validation and exhibits favorable clinical applicability.

**Table 3:** Performance comparison between ERA-CRISPR/Cas12a dual detection system and commercial qPCR kit.

		ERA-CRISPR/Cas12a Fluorescence System		ERA-CRISPR/Cas12a Lateral Flow System		Total (*n*=60)
		Positive	Negative	Positive	Negative	
qPCR	Positive	32	0	30	2	32
	Negative	0	28	0	28	28
	PPV/NPV	PPV: 100%	NPV: 100%	PPV: 93.8%	NPV: 100%	

## 4 Discussion

In recent years, there has been a consistent surge in the prevalence of infectious diseases worldwide, posing significant threats to human life and health. Before the onset of the novel coronavirus (COVID-19) pandemic, tuberculosis stood as the primary cause of mortality attributed to a single infectious agent, posing a risk of community transmission due to its highly contagious nature. As such, there is an urgent need for timely, rapid, and effective pathogen detection to prevent global public health crises. The CRISPR/Cas system, aside from its application in gene therapy, demonstrates potential for pathogenic nucleic acid detection, showcasing remarkable advantages in molecular diagnostics (Huang et al., 2022). The ultrasensitive and specific CRISPR/Cas12a system, combining polymerase-mediated DNA amplification detection enables the identification of various pathogens, including SARS-CoV-2 (Sun et al., 2021), *Mycoplasma pneumoniae* (Deng et al., 2022), *Porcine circovirus type 3* (Zhang et al., 2021), and *Porcine Parvovirus* (Wei et al., 2022). The objective of this study was to establish a novel detection method for the rapid and efficient identification of *M. tuberculosis*. In our method, ERA amplification and Cas digestion were performed in separate reaction tubes. Isothermal amplification facilitated exponential expansion of nucleic acid fragments, while Cas digestion further amplified the signal. Results from **Figure 2B** confirmed the compatibility and efficacy of CRISPR Cas12a with ERA.

Some strains of *M. tuberculosis*, particularly those prevalent in Asia, have been observed with limited or non-existent IS6110 insertion. According to Das et al., 40% of strains in South India, 29% in Thailand, and 20% in Vietnam contained only one copy of the IS6110 sequence (Das et al., 1995). The frequency of the IS6110 deletion strain (Beijing genotype ancient strain) ranged from 13.5% to 23.57% across different regions in China. Consequently, the presence of *M. tuberculosis* strains with reduced or absent IS6110 copies can markedly influence the positive rate and accuracy of detection methods that rely on IS6110 as the target. The *M. tuberculosis* IS1081 is capable of detecting all strains within this complex cluster, including those that lack the IS6110 gene (Chakravorty et al., 2017; Sharma et al., 2022). Notably, the IS1081 gene was selected for our investigation due to its relevance.

The ERA offers several advantages over qPCR. Firstly, the amplification process of ERA can be completed in 20 min, making it faster than qPCR. Secondly, ERA detection can be conveniently performed using a simple water bath and heating module, eliminating the necessity for expensive temperature control instruments (Liu et al., 2022). It is noteworthy that LAMP, another isothermal nucleic acid amplification technique with promising clinical applications, has been developed for the detection of *M. tuberculosis* (Wang et al., 2021). However, LAMP typically requires 4-6 pairs of primers to identify different regions of the target gene at a high temperature range of 60-65°C for efficient amplification. This complex primer design and potential primer hybridization may affect the specificity of amplification. The ERA not only requires a single pair of primers with relatively easy design but also operates at a reaction temperature ranging from 37 °C to 42°C, which is significantly lower than the temperature required by LAMP. MCDA has also been employed for the diagnosis of *M. tuberculosis* (Jia et al., 2023), however it suffers from certain drawbacks such as significant non-specific amplification leading to false positive results in blank control samples as well as sequence deviation. The difference between recombinant polymerase amplification (RPA) and ERA lies in their respective sources of recombinases-RPA utilizes T4 phage-derived recombinases while ERA employs recombinases derived from bacteria, viruses, and phages. Importantly, the ERA technology represents Chinese intellectual property providing a novel platform for molecular detection within China.

The newly developed ERA-CRISPR/Cas12a dual system demonstrates enhanced sensitivity and specificity for the detection of *M. tuberculosis*. **Table 4** summarizes various isothermal amplification methods combined with CRISPR/Cas12a protein for detecting *M. tuberculosis* (Ai et al., 2019; Xu et al., 2020; Li et al., 2021; Wang et al., 2021; Jia et al., 2023). It can be observed from **Table 4** that the fluorescence system exhibits higher sensitivity compared to other methods. The ERA-CRISPR/Cas12a dual system demonstrates exceptional timeliness and the shortest detection time, showcasing advantages in both sensitivity and speed of detection.

**Table 4:** Comparison of several detection methods for *M. tuberculosis*.

No.	Amplification methods and Cas enzymes	Dection method	LOD	Sensitivity	Specificity	Dection time(min)	Reference
1	ERA-Cas12a	F L	9 copies/µL 90 copies/µL	100% 93.8%	100% 100%	50 60	Our method
2	LAMP-Cas12a	F/L	10 copies/µL	79.50%	100%	60	Wang et al., 2021
3	MCDA-Cas12a	F/UV light	40 fg/recation	UR	100%	60	Jia et al., 2023
4	RPA-Cas12a	F	5 copies/µL	79%	98%	90	Ai et al., 2019
5	RPA-Cas12a	F	1 copies/µL	80%	100%	60	Li et al., 2021
6	RPA-Cas12a	F	4.48 fmol/L	99.29%	100%	4h	Xu et al., 2020

ERA, enzymatic recombinase amplification; LAMP, loop-mediated isothermal amplification; MCDA, multiple cross-displacement amplification; RPA, recombinase polymerase amplification; F, fluorescent; UV light, visual detection under UV light; L, Lateral flow test; UR, unreported.

To optimize the reaction conditions for maximum efficacy, we performed the selection of the reaction conditions for ERA amplification and carefully adjusted the concentrations of Cas proteins and reporter probes to maximize the assay’s effectiveness. We used the single-copy plasmid template by cloning the IS1081 target segment into the pUC57 plasmid. By utilizing this single-copy plasmid DNA, the ERA Fluorescence system achieved a limit of detection (LOD) of 9 copies/μL. The lateral flow system exhibited an LOD of 90 copies/μL. Furthermore, validation using standard strains and clinical samples demonstrated the excellent sensitivity and specificity of our detection system. The fluorescence system was consistent with the results of qPCR, and the PPV and NPV of clinical samples were 100%. The PPV and NPV of the lateral flow system were 93.8% and 100%, respectively (**Table 4**). Notably, compared to the qPCR, our study effectively minimized reaction time. The ERA amplification process required only 20 min, while Cas12a cleavage reaction took merely 10 min for real-time result analysis in conjunction with the fluorescence system that could determine positivity within just 30 min. In contrast, the Cas12a cleavage reaction in the lateral flow system necessitated a 15-min incubation period before test results could be read within 5 min, enabling completion of testing in a mere total time frame of approximately 40 min. Moreover, our method offers cost efficiency at approximately $7 per tube when compared to X-pert and qPCR alternatives. Additionally, this approach can be adapted for detecting other pathogens such as *Mycoplasma pneumoniae* through custom primers and gRNA (Deng et al., 2022). It also holds potential for identifying resistance genes associated with *M. tuberculosis*, including inhA and rpoB targets (Liu et al., 2021a; Bai et al., 2022).

The study, nevertheless, is subject to certain limitations. It is constrained by its exclusive focus on sputum samples, and a broader range of sample types should be included to further validate the diagnostic effectiveness of this testing method. Moreover, the sensitivity of the lateral flow system is comparatively lower than that of the fluorescence system due to inherent methodological constraints that need improvement. Additionally, it is crucial to note that the current system involves two separate steps: ERA isothermal amplification followed by CRISPR cleavage, which poses a risk of aerosol contamination during nucleic acid transfer. In order to mitigate potential aerosol pollution, we thoroughly wipe and disinfect the environment after each experiment. Subsequently, the area is exposed to ultraviolet light for 30 minutes to minimize the risk of aerosol contamination. Furthermore, continuous environmental monitoring is essential for timely detection of any aerosol contamination. The latest study by Peng et al. has demonstrated that the combination of CPA with CRISPR Cas12b enables one-pot detection of *M. tuberculosis*, offering valuable insights. Our future research will focus on exploring the feasibility of ERA and CRISPR Cas12a one-pot method, including optimizing the components of the CRISPR NEBffer2.1 reaction and incorporating liquid surfactants to enhance flow and reduce reaction viscosity.

This study combines ERA and CRISPR/Cas12a to establish an *M. tuberculosis*-DNA assay, which demonstrates rapidity, sensitivity, specificity, and has the capacity to address the challenges associated with molecular diagnosis in resource-limited environments. Additionally, our approach necessitates no supplementary equipment, and even a lateral-flow test strip can be employed for result interpretation. This confers utility to our system in the realms of diagnosis and screening, which is unattainable with PCR-based techniques. Therefore, our *M. tuberculosis* detection system can be effectively utilized in various fields, including clinical applications, for the accurate identification of pathogens.

## Data Availability

The original contributions presented in the study are included in the article/supplementary material. Further inquiries can be directed to the corresponding author.
